# Serum cystatin C and stroke risk: a national cohort and Mendelian randomization study

**DOI:** 10.3389/fendo.2024.1355948

**Published:** 2024-04-12

**Authors:** Yitian Qi, Xinyun Shang, Tianjiao Han, Ning Han, Ziwei Jiang, Han Yan, Siqi Yue, Qichao Sun, Lin Liu, Cancan Cui

**Affiliations:** ^1^ China-Japan Union Hospital of Jilin University, Jilin University, Changchun, Jilin, China; ^2^ The First Bethune Hospital of Jilin University, Jilin University, Changchun, Jilin, China

**Keywords:** cystatin C, stroke, EGFR, cohort study, Mendelian randomization

## Abstract

**Purpose:**

The debate over the causal and longitudinal association between cystatin C and stroke in older adults persists. Our aim was to assess the link between cystatin C levels, both measured and genetically predicted, and stroke risk.

**Methods:**

This study employed a retrospective cohort design using samples of the China Health and Retirement Longitudinal Study (CHARLS), which is a nationally representative cohort recruiting individuals aged 45 years or above. A multivariate logistic model and the two-sample Mendelian randomization framework were used to investigate the longitudinal and genetically predicted effect of serum cystatin C on stroke.

**Results:**

The study population had a mean age of 59.6 (SD ±9.5), with 2,996 (46.1%) women. After adjusting for confounding factors, compared to those in the first quartile of cystatin C, those in the last quartile had the greatest risk of stroke incidence [odds ratio (OR), 1.380; 95% confidence interval (CI), 1.046–1.825]. The Mendelian randomization analysis showed that a genetically predicted cystatin C level was positively associated with total stroke (OR by inverse variance-weighted method, 1.114; 95% CI, 1.041–1.192).

**Conclusions:**

This national cohort study suggests that higher serum cystatin C is associated with an increased risk of total stroke, which is further supported by Mendelian randomization.

## Introduction

Stroke is the leading cause of death and disability worldwide, and the global burden of stroke is significantly increasing (1). Globally, the absolute numbers of people with first stroke (16.9 million), stroke survivors (33 million), stroke-related deaths (5.9 million), and disability-adjusted life-years (DALYs) lost (102 million) were substantially high (2). Among the Chinese middle-aged and older population, there were 3.4 million incident cases of stroke, 17.8 million prevalent cases of stroke, and 2.3 million deaths from stroke (3). Thus, the early identification of potential risk factors and biomarkers is crucial to prevent or reduce the incidence of stroke and promote healthy aging.

A powerful inhibitor of lysosomal cysteine protease, cystatin C is employed in human vascular pathology, controlling cathepsins, and acting as a marker of renal activity (4, 5). Cathepsins may result in the remodeling and inflammation response of the vascular wall and are overexpressed in atherosclerotic lesions (6, 7). Notably, the association between cystatin C and stroke was highly contentious and inconsistent (8–10). A meta-analysis of nine cross-sectional studies found that patients of ischemic stroke had distinctly increased serum cystatin C concentrations compared to controls (11). Another cross-sectional study reported that higher cystatin C levels were directly associated with an increased proportion of stroke, including hemorrhagic and ischemic stroke (12). Data on prospective cohort studies are relatively limited and inconsistent. A study among the European population found that cystatin C concentrations were associated with ischemic stroke after adjusting for traditional risk factors (8). Contrary to what was found in a European population study, other studies have reported that cystatin C has no independent association with ischemic stroke or any type of stroke (10, 13, 14). Age distribution is a possible modification factor accounting for the heterogeneity in observational studies (15, 16). Moreover, it is reported that cystatin C is a possible determinant of endogenous neuroprotection and a protective factor against stroke in mechanism studies (17). Therefore, the longitudinal and causal relationship of cystatin C with stroke in the general population needs more evidence.

The purpose of this research was to assess the longitudinal correlation between serum cystatin C concentration and new-onset stroke, using a national cohort. Moreover, we employed a two-sample Mendelian randomization (MR) analysis to prevent any unmeasured confounding factors and reverse causation, thereby confirming the causal link between cystatin C and total stroke.

## Methods

### Study population

The current study was a secondary analysis using data from the China Health and Retirement Longitudinal Study (CHARLS), which is a national population-based cohort study (http://charls.pku.edu.cn/). A multistage stratified probability sampling strategy was employed to recruit participants from 150 counties or districts of 28 provinces in China, with biannual surveys conducted in 2011–2012 (as the baseline), 2013–2014, 2015–2016, and 2017–2018. Details of the study design and profile have been previously described (18). The CHARLS was approved by the Institutional Review Board of Peking University. During the surveys, data on sociodemographic features, anthropometric measures, lifestyles, and health information were gathered at each cycle. This study was conducted following the Strengthening the Reporting of Observational Studies in Epidemiology (STROBE) guideline.

**Figure 1:**
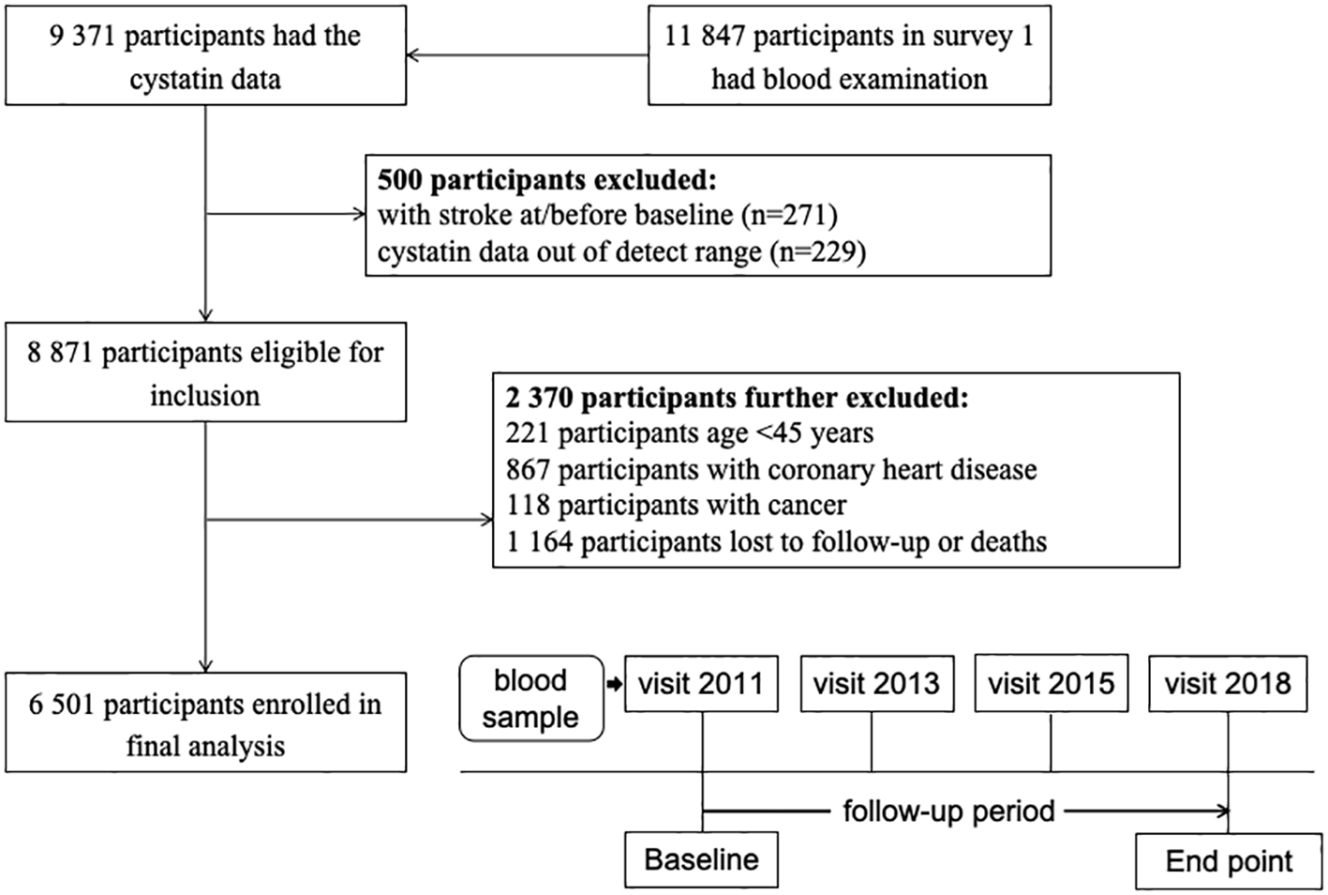
Flowchart and follow-up setting of the current study.

Our study was conducted using data from four surveys (2011, 2013, 2015, and 2018) of the CHARLS. In brief, of 11,847 participants with blood samples at the 2011 wave, 9,371 have available data on serum cystatin C levels. Then, 271 participants with stroke history were excluded. Those with cystatin C data out of the detection range were also excluded (*n* = 229). We also excluded participants younger than 45 years, those with coronary heart disease or cancer, and those without follow-up data. A total of 6,501 participants were included in the final analyses (**Figure 1**).

### Exposure and covariates

The Chinese Centre for Disease Control and Prevention collected and tested fasting venous blood samples. A particle-enhanced turbimetric assay was used to measure serum cystatin C (mg/L) with a coefficient of variation of less than 5%. Additionally, an immunoturbidimetric assay was used to measure high-sensitivity C-reactive protein (hs-CRP). Both assays of cystatin C and hs-CRP were conducted using the Hitachi 7180 chemistry analyzer (Hitachi, Japan). An enzymatic colorimetric test was utilized to gauge serum lipid markers and glucose levels, while non-HDL cholesterol was determined by subtracting HDL cholesterol from the total cholesterol concentration.

Questionnaires were utilized to acquire demographic information such as age, gender, place of residence, educational attainment, marital status, smoking status, current drinking, and past illnesses. Smoking status was divided into never, former, and current. Residence type was grouped into rural and urban. Education level was divided into primary, secondary, and tertiary. Marital status was grouped into married and others. Using anthropometric measurements, body mass index (BMI) was calculated as the ratio of weight (kilograms) to height (meters) squared. Obesity was determined to be a BMI of more than 28.0, exclusive to the Chinese population. Blood pressures were presented as the average of measurements. Hypertension was defined as systolic pressure ≥140 mmHg, diastolic pressure ≥90 mmHg, or a self-reported diagnosis history of hypertension or using any anti-hypertensive medication (19). Diabetes was defined as fasting glucose  ≥ 7.0 mmol/L, self-reported diagnosis history of diabetes, or using any glucose-lowering medication according to the American Diabetes Association (20).

### Definition of incident stroke

The study outcome was incidence of stroke during the follow-up period (from wave 2 to wave 4). As described previously (21), information on the diagnosis of stroke was collected using the standardized question: “Have you been told by a doctor that you have been diagnosed with a stroke?” Those without a history of stroke attack prior to the baseline survey and reported a diagnosis of stroke during the follow-up period were considered as stroke incidence. Standardized questionnaires, complemented with international leading aging surveys, such as the Health and Retirement Study (HRS), English Longitudinal Study of Ageing (ELSA), and Survey of Health, Aging and Retirement in Europe (SHARE), were used to evaluate the outcomes. To guarantee data reliability, a stringent quality control and accuracy checking process was conducted (22).

### Data of Mendelian randomization

A two-sample MR technique was employed to ascertain the causal influence of cystatin C on the risk of stroke, in agreement with the prior observational study. The genetic variants used must satisfy three assumptions: (1) the genetic variants used as instrumental variables (IVs) are strongly associated with cystatin C; (2) the genetic variants are not associated with other confounders; and (3) the genetic variants are associated with stroke exclusively through cystatin C. The data on the interest of exposure (cystatin C) were derived from a meta-analysis GWAS of 363,228 individuals (23). The genetic data for stroke were obtained from a summarized database released by the MEGASTROKE project by the International Stroke Genetics Consortium (24), including 406,111 controls and 40,585 stroke cases.

### Statistical analysis

The mean [standard deviation (SD)] and number (proportion) of continuous and categorical variables were used to present baseline characteristics according to quartile groups of serum cystatin levels.

To determine the association between serum cystatin C and incident stroke, multivariable-adjusted logistic regression models were used to calculate the odds ratio (OR) with 95% confidence interval (CI). Cystatin C levels were evaluated as both continuous variables (log transformed due to skewed distribution) and quartiles, with the lowest quartile serving as the reference group. The normal distribution was tested using the Kolmogorov–Smirnov (K–S) method. Individual-level factors were adjusted step by step in two models: Model 1 was adjusted for age groups (<65, 65–84, and ≥85) and sex; Model 2 was further adjusted for residence (rural and urban), education level (primary, secondary, and third), marital status (married and others), smoking status (current, former, and never), current drinking (yes and no), obesity (yes and no), hypertension (yes and no), diabetes (yes and no), triglyceride (continuous), non-HDL cholesterol (continuous), and glucose (continuous). The missingness of covariates adjusted in the regression model was treated as a category of “missing” in the main analysis. A restricted cubic spline function, with 3 knots at the 10th, 50th, and 90th percentiles, was employed to analyze the dose–response correlation between serum cystatin C and stroke risk. The 10th value of cystatin C was used as the reference point.

We performed multiple sensitivity analyses after additionally adjusting for hs-CRP level (continuous) or blood sample fasting status (yes and no) in separate models. Markov chain Monte Carlo was employed to execute multiple imputed analyses for missing data (five iterations), and the pooled results were summarized. The number of each missing variable is shown in the **Supplementary Materials** (**Table S2**). Moreover, all regression analyses were repeated among subgroups in terms of age, sex, obesity, residence, smoking, hypertension, and diabetes.

For two-sample MR analysis, we selected single-nucleotide polymorphisms (SNPs) previously shown to be associated with the cystatin C trait at the level of genome-wide significance (*p* < 5×10^−8^). To avoid linkage disequilibrium (LD) among IVs, we calculated the LD parameter (*r* ^2^) between SNPs based on the reference panel consisting of 1000 Genomes Project European sample data. We assessed the independence of SNPs using stringent criteria (*r* ^2^<0.001; clumping window, 10,000 kb). MR analysis relies on three key assumptions of IVs. The first assumption is that IVs should be strongly associated with the exposure; the second assumption is that IVs should be independent of any confounders; and the third assumption is that IVs should only affect the outcome through the exposure and not through other pathways (25). Using the summary statistics of effect sizes of each instrumental SNP with exposure and outcome (dichotomous), the harmonization of the direction was estimated by effect alleles. The Wald estimator was employed to calculate the effect for each instrument, and the Delta method was used to calculate the standard errors. Subsequently, MR estimates were pooled to infer the causal effect of exposure on stroke, utilizing random inverse variance-weighted meta-analysis and Pleiotropy RESidual Sum and Outlier (PRESSO) due to the existing heterogeneity and horizontal pleiotropy.

R software (version 4.1.0) was utilized for all statistical analyses, and a two-sided *p*-value of less than 0.05 was deemed statistically significant.

## Results

### Baseline characteristics

As illustrated in the flowchart (**Figure 1**), a total of 6,501 participants were included in the analyses. The mean (SD) age was 59.6 (9.5) years, and 2,996 (46.1%) were women. **Table 1** summarizes the characteristics according to baseline cystatin C quartiles. Participants with higher serum cystatin C level were older, more likely to be men, and current drinkers.

**Table 1:** Baseline characteristics of 6,501 participants according to baseline cystatin C levels.

	Overall	Quartile 1	Quartile 4
Participants, *N*	6,501	1,640	1,557
Age, years, mean	59.57 (9.52)	55.18 (8.06)	66.03 (9.68)
Sex, male, *n* (%)	2,996 (46.1)	530 (32.3)	925 (59.4)
Residence, *n* (%)			
Rural	5,225 (80.4)	1,293 (78.9)	1,237 (79.4)
Urban	1,272 (19.6)	345 (21.1)	320 (20.6)
Marital status, married, *n* (%)	5,731 (88.2)	1,499 (91.4)	1,270 (81.6)
Educational level, *n* (%)			
Primary	4,460 (68.7)	1,050 (64.1)	1,165 (74.9)
Secondary	1,302 (20.0)	378 (23.1)	243 (15.6)
Third	734 (11.3)	210 (12.8)	147 (9.5)
Smoking status, *n* (%)			
Never	3,896 (60.0)	975 (59.6)	932 (59.9)
Former	456 (7.0)	109 (6.7)	105 (6.8)
Current	2,140 (33.0)	552 (33.7)	518 (33.3)
Current drinking, *n* (%)	2,251 (34.7)	549 (33.6)	566 (36.4)
BMI, kg/m^2^, *n* (%)			
<23.9	3,027 (56.5)	750 (55.7)	740 (56.7)
24–27.9	1,137 (21.2)	299 (22.2)	274 (21.0)
≥28	1,196 (22.3)	297 (22.1)	292 (22.4)
SBP, mmHg, mean	131.13 (21.97)	131.62 (22.26)	131.27 (22.40)
Hypertension, *n* (%)	2,772 (42.6)	714 (43.5)	666 (42.8)
Diabetes, *n* (%)	1,199 (18.4)	348 (21.2)	281 (18.0)

Data are presented as mean (SD) or number (%), as appropriate.

SD, standard deviation; BMI, body mass index; SBP, systolic blood pressure.

a Calculated as weight in kilograms divided by height in meters squared.

### Longitudinal association between cystatin C and stroke

**Figure 2:**
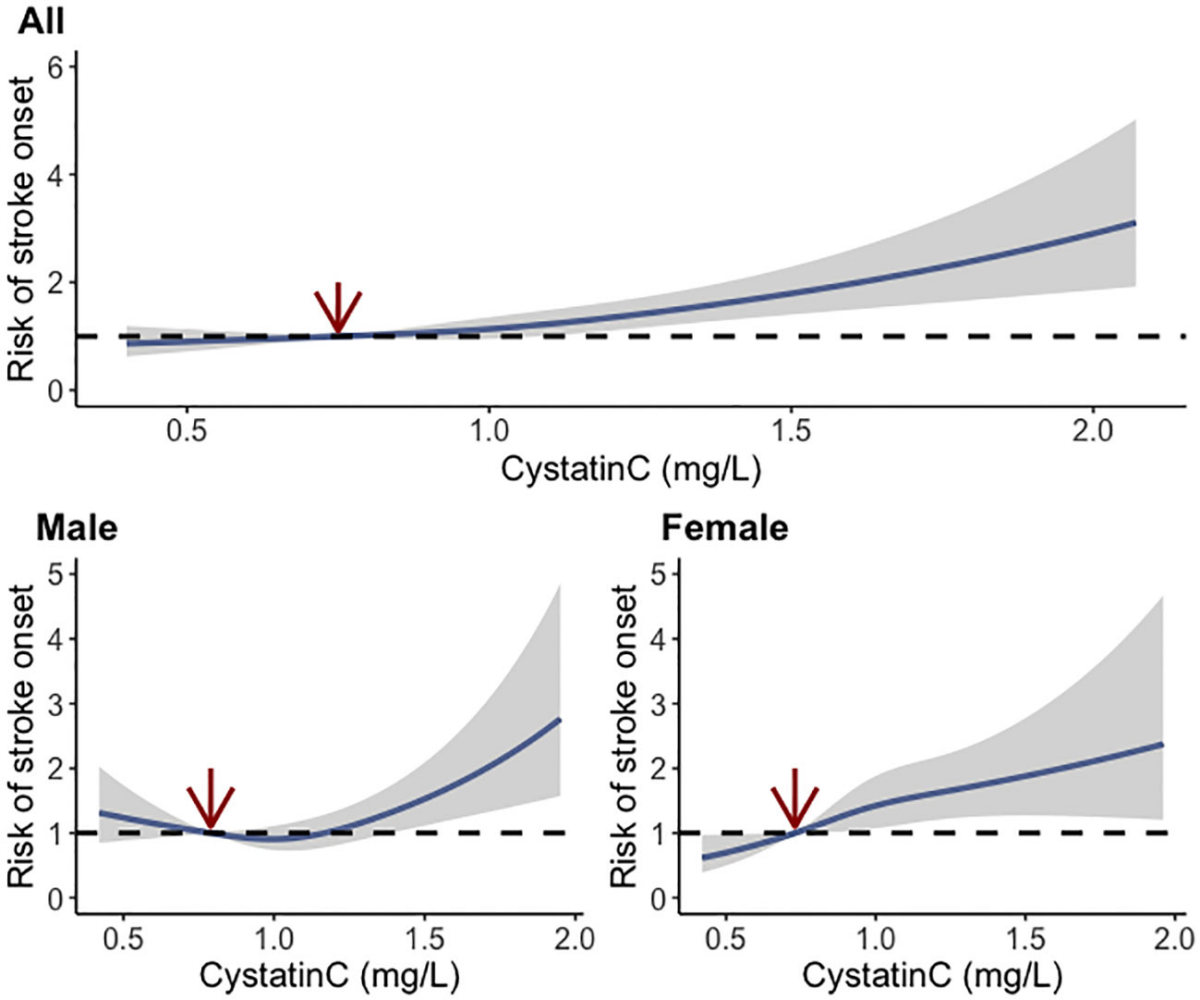
Dose–response relationship between baseline cystatin C and risk of stroke using the restricted cubic spline method.

During a follow-up up to 7.0 years, 651 (10.0%) cases developed stroke events, and the incidence rates were 7.9%, 9.4%, 9.4%, and 13.2% among cystatin C quartile groups, respectively. The trends of increased stroke risk concordant with a higher cystatin C level were similar across male and female participants (**Figure 2**). In the fully adjusted model, there was a positive association between serum cystatin C concentration and new-onset stroke (**Table 2**). Compared with people of the first quartile of cystatin C, those in the last quartile had the highest risk of stroke (OR, 1.380; 95% CI, 1.046–1.825) when cystatin C was assessed as quartiles. Consistent results were observed among multiple sensitivity analyses when additionally adjusting for hs-CRP and blood fasting status (**Supplementary Table S1**) and using imputed data (**Supplementary Table S2**). Moreover, cystatin C outperformed serum creatinine (also an important indicator of renal function) in terms of the discriminative capacity of stroke (AUC: 55.6% vs. 54.0%) as shown in **Supplementary Figure S1**. The difference in AUC values was statistically significant per DeLong’s test (*p* = 0.015). The associations between cystatin C and incident stroke remained in subgroups stratified by age, sex, obesity, residence, smoking, hypertension, and diabetes (**Table 3**). Of note, the association between cystatin C and stroke seemed stronger among female participants, and the adjusted ORs for participants in the second, third, and fourth quartile were 1.505 (95% CI, 1.012–2.258), 1.643 (95% CI, 1.110–2.457), and 1.743 (95% CI, 1.158–2.647), respectively.

**Table 2:** Associations of baseline cystatin C and the risk of stroke.

	No. of stroke/Total	Model 1		Model 2	
		OR (95% CI)	*p*	OR (95% CI)	*p*
Log (cystatin C) (per unit)	–	1.584 (1.060–2.371)	0.025	1.604 (1.025–2.515)	0.039
Quartile 1 (0.40–0.85)	129/1,640	Ref		Ref	
Quartile 2 (0.86–0.97)	163/1,674	1.217 (0.956–1.554)	0.112	1.176 (0.901–1.539)	0.237
Quartile 3 (0.98–1.11)	153/1,630	1.105 (0.861–1.420)	0.435	1.151 (0.878–1.512)	0.310
Quartile 4 (1.12–2.45)	206/1,557	1.486 (1.156–1.915)	0.002	1.380 (1.046–1.825)	0.023

OR, odds ratio; CI, confidence interval.

Model 1 was adjusted for age group and sex; model 2 was further adjusted for residence, marital status, education level, BMI group, smoking status, current drinking, hypertension, diabetes, triglyceride, non-HDL cholesterol, and glucose.

**Table 3:** Subgroup analysis of associations between baseline cystatin C quartile and the risk of stroke.

Subgroups	OR (95% CI), *p*-value		
	Quartile 2	Quartile 3	Quartile 4
Sex			
Male	0.847 (0.598–1.196), 0.347	1.129 (0.821–1.556), 0.455	1.490 (1.095–2.034), 0.012
Female	1.505 (1.012–2.258), 0.045	1.643 (1.11–2.457), 0.014	1.743 (1.158–2.647), 0.008
*p*_for interaction_	0.011	0.082	0.120
Age			
<65 years	1.248 (0.926–1.686), 0.148	1.234 (0.91–1.678), 0.176	1.423 (1.056–1.925), 0.021
>65 years	1.084 (0.697–1.693), 0.721	1.279 (0.823–1.996), 0.276	1.57 (1.022–2.432), 0.041
*p*_for interaction_	0.585	0.566	0.760
Obesity			
No	1.722 (1.213–2.462), 0.003	1.336 (0.918–1.953), 0.131	1.719 (1.182–2.516), 0.005
Yes	0.782 (0.413–1.451), 0.441	1.335 (0.777–2.313), 0.297	1.646 (0.974–2.819), 0.065
*p*_for interaction_	0.058	0.978	0.951
Residence			
Rural	1.118 (0.859–1.456), 0.407	1.046 (0.802–1.364), 0.742	1.312 (1.002–1.721), 0.049
Urban	1.454 (0.788–2.743), 0.236	1.319 (0.7–2.527), 0.395	2.361 (1.27–4.524), 0.008
*p*_for interaction_	0.895	0.902	0.351
Smoking			
Never	1.099 (0.804–1.505), 0.554	1.098 (0.799–1.512), 0.566	1.406 (1.017–1.951), 0.04
Current/quit	1.411 (0.962–2.085), 0.08	1.109 (0.741–1.667), 0.615	1.614 (1.086–2.417), 0.019
*p*_for interaction_	0.476	0.541	0.352
Hypertension			
No	1.025 (0.737–1.424), 0.884	1.028 (0.741–1.425), 0.868	1.615 (1.191–2.199), 0.002
Yes	1.559 (1.089–2.245), 0.016	1.382 (0.953–2.015), 0.089	1.87 (1.279–2.752), 0.001
*p*_for interaction_	0.005	0.191	0.059
Diabetes			
No	1.158 (0.881–1.522), 0.293	1.034 (0.785–1.362), 0.814	1.406 (1.066–1.859), 0.016
Yes	1.044 (0.6–1.818), 0.878	1.436 (0.862–2.422), 0.168	1.819 (1.096–3.064), 0.022
*p*_for interaction_	0.591	0.425	0.862

OR, odds ratio; CI, confidence interval.

Analyses were adjusted for age group, sex, residence, marital status, education level, BMI group, smoking status, current drinking, hypertension, diabetes, triglyceride, non-HDL cholesterol, and glucose if not stratified using the lowest quartile as reference group.

### Two-sample MR analysis

There were 223 SNPs selected to infer the causal relationship between cystatin and stroke. Heterogeneity for MR analysis was represented by Cochran *Q* (*p*-value, 9.685×e^−9^), and thus, random-effect models were used. The inverse variance-weighted models showed that the genetically predicted one-SD increase of cystatin C was associated with a higher risk of lifetime stroke [OR, 1.114 (95% CI, 1.041–1.192); *p* = 0.002; **Table 4**]. We did not detect any potential pleiotropy effect (MR-Egger intercept, 0.003; *p* = 0.138). The causal association of cystatin C with stroke remained after removing three outliers (pleiotropic SNPs) using MR-PRESSO [OR, 1.128 (95% CI, 1.058–1.203); *p* < 0.001].

**Table 4:** Mendelian randomization estimates for the association of cystatin C with stroke using the inverse variance-weighted (IVW) model and Pleiotropy RESidual Sum and Outlier (PRESSO).

Outcome	Methods	Cystatin C		
		OR	95% CI	*p*-value
Stroke	IVW	1.114	1.041–1.192	0.002
	PRESSO	1.128	1.058–1.203	<0.001
Heterogeneity	Cochran *Q*			9.685×e^−9^
Pleiotropy	MR-Egger			0.138

## Discussion

In the current analysis using a national cohort, we found that cystatin C level is significantly associated with new-onset stroke event among the Chinese population. Cystatin C is a potential indicator for stratifying the risk of stroke compared to serum creatinine. Moreover, the two-sample MR analysis showed that higher genetically predicted cystatin C level is causally associated with a higher risk of stroke. The findings suggested that cystatin C measurement should be incorporated into the assessment of stroke risk from the aspect of renal function (26), which could also be a possible target for improving cardiovascular health.

The association between cystatin C and stroke has been investigated in previous studies. The positive associations between cystatin C and stroke or cardiovascular risk have also been reported among the European, US, Chinese, and multi-ethnic populations (10, 27–30). When compared with serum creatinine, a study of the elderly community population found that cystatin C is a stronger predictor of death and cardiovascular events compared to serum creatinine (9). Another study involving 4,650 middle-aged subjects reported that cystatin C is a better indicator for cardiovascular health than glomerular filtration rate (GFR) calculated using creatinine (5, 31). A study among asymptomatic carotid atherosclerosis patients even claimed that cystatin C was significantly associated with subsequent cardiovascular events and stroke but not serum creatinine or estimated GFR (32). In contrast, there are other studies showing that there is no significant association between cystatin C and cardiovascular diseases including stroke (13, 33), indicating more inconsistency regarding the relationship between cystatin C and stroke. A cross-sectional study reported that serum cystatin C levels were associated with higher risks of both hemorrhagic and ischemic stroke and the prognosis of stroke patients (12). A prospective pooled analysis using six cohorts summarized that cystatin C is significantly associated with ischemic stroke (8). Similar results were observed in terms of the high risk of stroke (34) or the severity of stroke (35). In a community-based population, the authors reported the association between serum cystatin C and cerebral small vessel disease (36). In contrast, other studies reported that cystatin C was not independently associated with ischemic stroke or any type of stroke (13, 14). In a cohort study, the significance of the relationships between cystatin C and stroke onset was switching when different cutoff values were used (21), which is partially due to over-adjusting. Moreover, there is existing evidence about the relationship between cystatin C and prognosis among stroke patients (37–39). The demographic characteristics of previous studies (such as age distribution, sex proportion, and race) could possibly account for the substantial heterogeneity. The Reasons for Geographic and Racial Differences in Stroke (REGARDS) cohort strongly suggested that the association of risk factors with stroke differed by race and sex (40), highlighting the need for validation regarding cystatin C and stroke on various races and populations. Our study supported the evidence that cystatin C is a predictor of new-onset stroke among Chinese adults, and the results were consistent after controlling for important risk factors. In addition, cystatin C outperformed serum creatinine regarding the discrimination capacity of stroke. The subgroup analysis showed that the effects of cystatin C were not strongly modified by age, BMI, smoking, hypertension, or diabetes status. The fact is that the effect seemed more stronger in female participants in our analysis, which is also noted in another study reporting that cystatin C was only a risk factor for all-cause mortality in female participants (13).

Regarding the MR analysis, previous studies have yielded inconsistent evidence in terms of the causal association between cystatin C and stroke. A study using one common variant, rs911119, in the CST3 gene as an IV did not find a causal effect of cystatin C on stroke (8). A one-sample MR study using UK Biobank data reported that there is no causal association between cystatin C and stroke, which is opposite to the observational findings (41). However, a recent genetics study identified the loci associated with serum cystatin C among 363,228 individuals and hinted on the causal effect on stroke (23). Our study is based on a two-sample MR design to take advantage of the maximum sample size and sufficient statistical power to infer the causality. The findings supported the causal association between cystatin C and total stroke. The different MR designs and sample sizes, especially for the stroke case number, could partially account for the distinct results.

Given the longitudinal and causal effects of cystatin C on the risk of stroke, the monitoring of cystatin C beyond the routine measurement of creatinine could provide additional information on the risk stratification and prediction of stroke. There are increasing cost-effective interventions from the public health perspective that targeting the risk factors in the population potentially has a substantial impact on reducing stroke burden (42). Apart from addressing traditional well-known factors (such as tobacco use, unhealthy diet, and hypertension), cystatin C monitoring from the kidney function aspect provides another risk enhancer accounting for the residual risk of stroke.

Several limitations of the current study should be acknowledged. First, the disease diagnosis history in the CHARLS was self-reported. However, it has been reported that self-reported cardiovascular diseases were highly consistent with medical records (43, 44). Second, the outcome in this study is diagnosis history of any stroke. The effects of cystatin C on subtypes of stroke could be distinct, which needs further elaboration. Third, the cohort study is conducted in Chinese participants aged 45 years and older, while the MR analysis retrieved data from the European population. The findings may not be fully generalized to each other. Overall, the causal relationship between cystatin C and stroke or its subtypes warrants more evidence from both epidemiologic and genetic perspectives.

In summary, this study supported the causal association between serum cystatin C level and the risk of stroke, combining the national cohort design and two-sample MR analysis. The underlying mechanism and the potential clinical benefit targeting cystatin C warrant further research.

## Data availability statement

The original contributions presented in the study are included in the article/**Supplementary Material**. Further inquiries can be directed to the corresponding authors.

## Ethics statement

The studies involving humans were approved by the Institutional Review Board of Peking University. The studies were conducted in accordance with the local legislation and institutional requirements. Written informed consent for participation in this study was provided by the participants’ legal guardians/next of kin.

## Author contributions

YQ: Writing – original draft. XS: Writing – original draft. TH: Writing – review & editing. NH: Writing – original draft. ZJ: Writing – review & editing. HY: Writing – original draft. SY: Writing – review & editing. QS: Writing – review & editing. LL: Writing – review & editing. CC: Writing – original draft, Writing – review & editing.
