# Supplementary material for: Serum cystatin C and stroke risk: a national cohort and Mendelian randomization study
Source: Front Endocrinol (Lausanne). 2024 Apr 12;15:1355948. doi: 10.3389/fendo.2024.1355948 (PMC11045987; doi:10.3389/fendo.2024.1355948)
Supplement: Supplementary file 1 [file DataSheet_1.docx]

**Supplemental Material**

**Association of cystatin C and risk of stroke: a national cohort and Mendelian randomization study**

**Supplemental Tables and Figures**

**Table S1: Sensitivity analyses of the association between cystatin C and the risk of stroke.**

|  | adjusted OR (95% CI), P value | |
| --- | --- | --- |
|  | Sensitivity A | Sensitivity B |
| Quartile 1 (0.40-0.85) |  |  |
| Quartile 2 (0.86-0.97) | 1.176(0.901-1.539), 0.237 | 1.171(0.895-1.536), 0.251 |
| Quartile 3 (0.98-1.11) | 1.151(0.878-1.512), 0.310 | 1.112(0.845-1.466), 0.451 |
| Quartile 4 (1.12-2.45) | 1.380(1.045-1.826), 0.024 | 1.385(1.048-1.836), 0.023 |

Abbreviation: OR, odds ratio; CI, confidence interval; SBP, systolic blood pressure.

Analyses were adjusted for age group, sex, residence, marriage, education level, BMI group, smoking status, current drinking, hypertension (yes/no), diabetes, triglyceride (continuous), nonHDL cholesterol (continuous), glucose (continuous).

Sensitivity A: hs-CRP additionally adjusted; Sensitivity B: blood sample fasting status additionally adjusted.

**Table S2:** **Pooled association between cystatin C and the risk of stroke using five imputed data.**

|  | OR | 95% CI | P value |
| --- | --- | --- | --- |
| Cystatin C (1.0 mg/L) | 1.689 | 1.161-2.456 | 0.006 |
| Quartile 1 (0.40-0.85) | Ref |  |  |
| Quartile 2 (0.86-0.97) | 1.249 | 0.978-1.596 | 0.075 |
| Quartile 3 (0.98-1.11) | 1.130 | 0.878-1.456 | 0.343 |
| Quartile 4 (1.12-2.45) | 1.527 | 1.183-1.971 | 0.001 |

Abbreviation: OR, odds ratio; CI, confidence interval.

Analyses were adjusted for age group, sex, residence, marriage, education level, BMI group, smoking status, current drinking, hypertension (yes/no), diabetes, triglyceride (continuous), nonHDL cholesterol (continuous), glucose (continuous).

Multiple imputation (five iterations) was performed using Markov chain Monte Carlo method for BMI (missing n=150), smoking or drinking status (missing n=9), education level (missing n=5), residence (missing n=4).


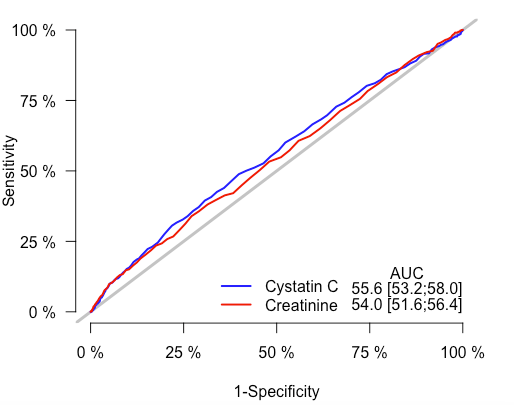


**Figure S1: Discrimination capacity between cystatin C and creatinine for stroke onset.**
